# Tolerance towards gentamicin is a function of nutrient concentration in biofilms of patient-isolated *Staphylococcus epidermidis*

**DOI:** 10.1007/s12223-017-0568-x

**Published:** 2017-11-22

**Authors:** Christoph G. Ammann, David Neuhauser, Claudia Eberl, Michael Nogler, Débora Coraça-Huber

**Affiliations:** 1 Experimental Orthopaedics, Medical University of Innsbruck, Innrain 36, 6020 Innsbruck, Austria; 2 Division of Hygiene and Medical Microbiology, Medical University of Innsbruck, Schöpfstraße 41, Innsbruck, Austria; 3 University Hospital for Orthopaedics, Anichstraße 35, Innsbruck, Austria

## Abstract

*Staphylococcus epidermidis* is a biofilm-forming bacterial strain that can cause major problems as an agent of nosocomial infections. Bacteria in biofilms are shielded from the environment and can survive high doses of antibiotics. We here test the antibiotic susceptibility of *Staphylococcus epidermidis* to rising gentamicin concentrations in optimal growth conditions as used in routine bacteriology laboratories with low nutrient situations as suggested to be found in clinical situations. We found that gentamicin-resistant *Staphylococcus epidermidis* biofilms survived in the absence of external nutrient supply in PBS. While addition of gentamicin sulfate significantly reduced the pH value of all used media and solutions, this acidification did not alter survival of bacteria in the biofilm. We found a statistically significant and dose-dependent reduction of survival in low nutrient situations using gentamicin sulfate in three out of four patient isolates of *Staphylococcus epidermidis* which have been tested to be gentamicin-resistant under optimal growth conditions. Supporting the original profiling, survival in full media under the same antibiotic dosages was not significantly reduced. Our data here show that antibiotic resistance is a function of the provided nutrient concentration. Antibiotic resistance profiling should consider variations in nutrient availability.

## Introduction

*Staphylococcus epidermidis* is a bacterial strain causing significant problems in nosocomial, hospital-acquired infections (Stegger et al. 2014; Gomes et al. 2014; Costerton et al. 1999; Harris and Richards 2006). *Staphylococcus epidermidis* is a common resident of the human bacterial flora on skin and mucosal surfaces (Kluytmans et al. 1997). When transferred to biological niches in the patient, these bacteria can attach to surfaces and have the capability to subsequently build up biofilms (Otto 2008). These multicellular conglomerates form in stages from attachment of single bacteria (Clarke and Foster 2006), through massive proliferation and quorum sensing-activated maturation. Quorum sensing is a form of communication between bacteria to determine culture size (Davies et al. 1998). Once a sufficient number of bacteria is reached, the biofilm shields itself from the surroundings by the expression of an extracellular matrix (Branda et al. 2005; Sutherland 2001). This extracellular matrix contains water, peptides, proteins, nucleotides, and sugars (Joo and Otto 2012). All these factors can be recycled by the bacteria in times of limited nutrient supply from external sources. Mature biofilms not only feature a three-dimensional network of tunnels allowing influx and efflux of solutions and soluble particles but also allow direct bacteria to bacteria contact.

It has been shown that bacteria in a biofilm can display phenotypes generally different from the planktonic growth phase (Stewart and Costerton 2001). This change in phenotype also enables tolerance to antibiotics in an up to thousand-fold higher concentrations compared to the tolerating capacity of free-floating bacteria (Lewis 2001). The so-called biofilm inhibitory concentration (BIC) has to be viewed as completely independent from the minimal inhibitory concentration (MIC) of planktonic cells (Coraça-Huber et al. 2012). To this study, it is important to note that the MIC and BIC are routinely tested under optimal growth conditions in full medium and that the nutrient saturation and availability in the in vivo setting differ significantly, especially in cases of clinical manipulation, e.g., after prosthetic joint surgery (Konttinen et al. 2001). Together, these biofilm-specific features enable bacteria to persist in the host.

We here combine testing of two attributes of biofilms simultaneously, which are the survival in low nutrient saturations and the high tolerability towards antibiotics.

## Material and methods

### Bacterial strains

*Staphylococcus epidermidis* ATCC 12228 was incubated at 37 °C on a Müller Hinton agar dish. Colonies were picked and a solution of 0.5 McFarland turbidity (approximately 10^8^ bacteria per mL) (Zapata and Ramirez-Arcos 2015) was generated in full Müller Hinton broth.

Patient isolates (PI) were derived from surgically removed infected prosthetic joints by sonication in an ultrasound water bath (41 kHz, Bandolin Bactosonic, Berlin, Germany) for 1 min in sterile phosphate-buffered saline (PBS). Sonication fluid (100 μL) was spread onto Müller Hinton agar dishes and after 48 h, single colonies were picked and grown as described above for the ATCC strains. Gentamicin resistance was determined at the Department of Hygiene, Microbiology and Social Medicine, Division of Hygiene and Medical Microbiology, Medical University of Innsbruck, Innsbruck, Austria, by EUCAST disc diffusion test.

### Media and growth conditions

Full Müller Hinton medium (MH) was diluted with sterile PBS to generate the following concentrations: 100% MH, 50% MH, 25% MH, and 0% MH. Single wells were inoculated with 1 mL of a 0.5 McFarland bacterial solution (approximately 10^8^ bacteria) and placed in a moist chamber (MC). This MC consists of water-wetted paper towels placed underneath the tissue culture plate (48-well format, plastic, Cellstar, Greiner Bio-One GmbH, Kremsmünster, Austria) in a sealable plastic container. Biofilms were grown at 37 °C in full MH for 48 h. The lid was closed thoroughly to generate a closed system reducing evaporation of culture media in exchange with the saturated humidified atmosphere surrounding it. One representative of two independent experiments is shown.

### pH

pH values of the used solutions in duplicates were determined using an electronic pH meter (Inolab 720, WTW, Weilheim, Germany). If necessary, pH was adjusted using sulfuric acid. All solutions were sterile filtered using a 0.22-μm filter after determination of the pH value.

### Survival test

Forty-eight-hour biofilms of *Staphylococcus epidermidis* were washed thoroughly with sterile PBS to remove planktonic cells from the culture plate. Subsequently, 1 mL of media with the indicated different pH values or different concentrations of gentamicin sulfate (Heraeus Medical GmbH, Germany) was added and the biofilm was cultivated for 24 h under these conditions. Gentamicin concentrations (10-fold serial dilutions of GS from 0.00005 mg gentamicin per mL to 0.5 mg gentamicin per mL) were prepared regarding the gentamicin base calculating out the sulfate residue. After three washing steps, 1 mL of sterile PBS was added to each well and the plate sonicated at 41 kHz for 1 min in an ultrasound sonication water bath. Ten microliters of the solution containing the detached surviving bacteria was spread onto Müller Hinton agar plates and incubated at 37 °C for 24 h. Bacterial counts were thus determined with a 100 cfu/mL detection limit. Two independent sets of experiments were performed.

### Passaging

Bacterial biofilms were grown for 48 h on and bacterial counts were determined after ultrasonic treatment as mentioned above. Single colonies of equal size were picked and diluted in 1 mL of PBS. Ten microliters of this solution was spread on Müller Hinton agar plates and colony counts were determined after 24 h of incubation at 37 °C. This procedure was repeated to determine if all tested strains could reach equal colony counts when grown under optimal growth conditions. Experiments were performed in quadruplicates.

### Statistical analysis

Data were analyzed using GraphPad Prism 6 software. *T* test was used to compare two groups of values. One-way ANOVA was used to compare multiple groups. Two-way ANOVA was used to compare grouped values. Dunnett’s multiple comparison procedure was used to determine differences of multiple groups compared to one control group. *p* values below 0.05 were considered statistically significant.

## Results

### Nutrient-dependent survival of *Staphylococcus epidermidis*

**Fig. 1:**
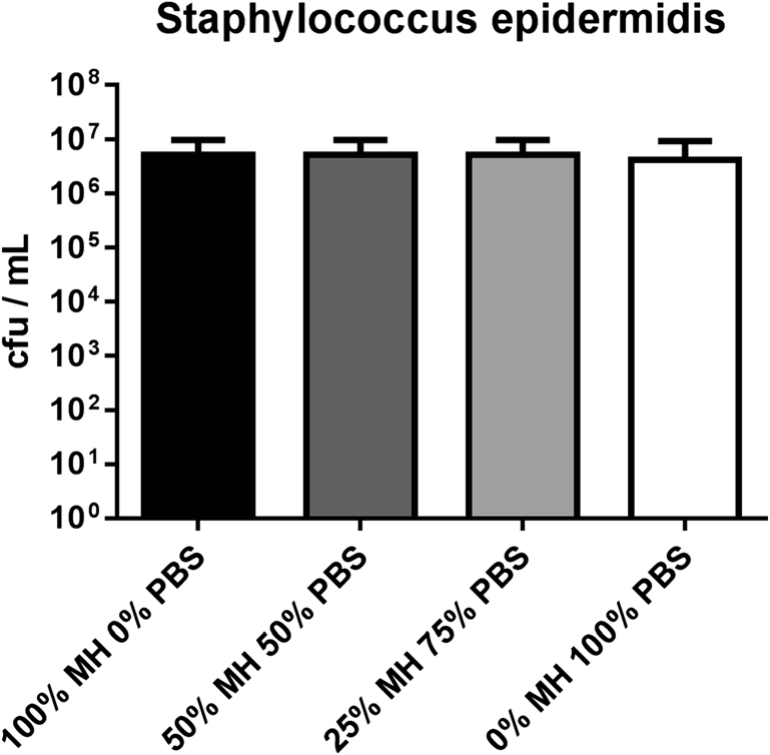
Nutrient-dependent survival of 48-h *Staphylococcus epidermidis* biofilms in varying growth medium concentrations. Pooled data of four clinical patient isolate strains and one ATCC strain

We were first interested in determining the survival of *Staphylococcus epidermidis* isolates in media with low nutrient concentration. We grew biofilms of four patient isolates and *Staphylococcus epidermidis* ATCC 12228 for 48 h and subjected the biofilms to dilutions of full Müller Hinton media and PBS. *Staphylococcus epidermidis* showed no reduction in survival under lowered nutrient concentration (Fig. 1); bacterial counts were unaltered by reduction of the nutrient concentration in any of the four tested growth media (100% MH, 50% MH, 25% MH, and 0% MH).

### pH of gentamicin solutions and pH-dependent survival of *Staphylococcus epidermidis* isolates

**Fig. 2:**
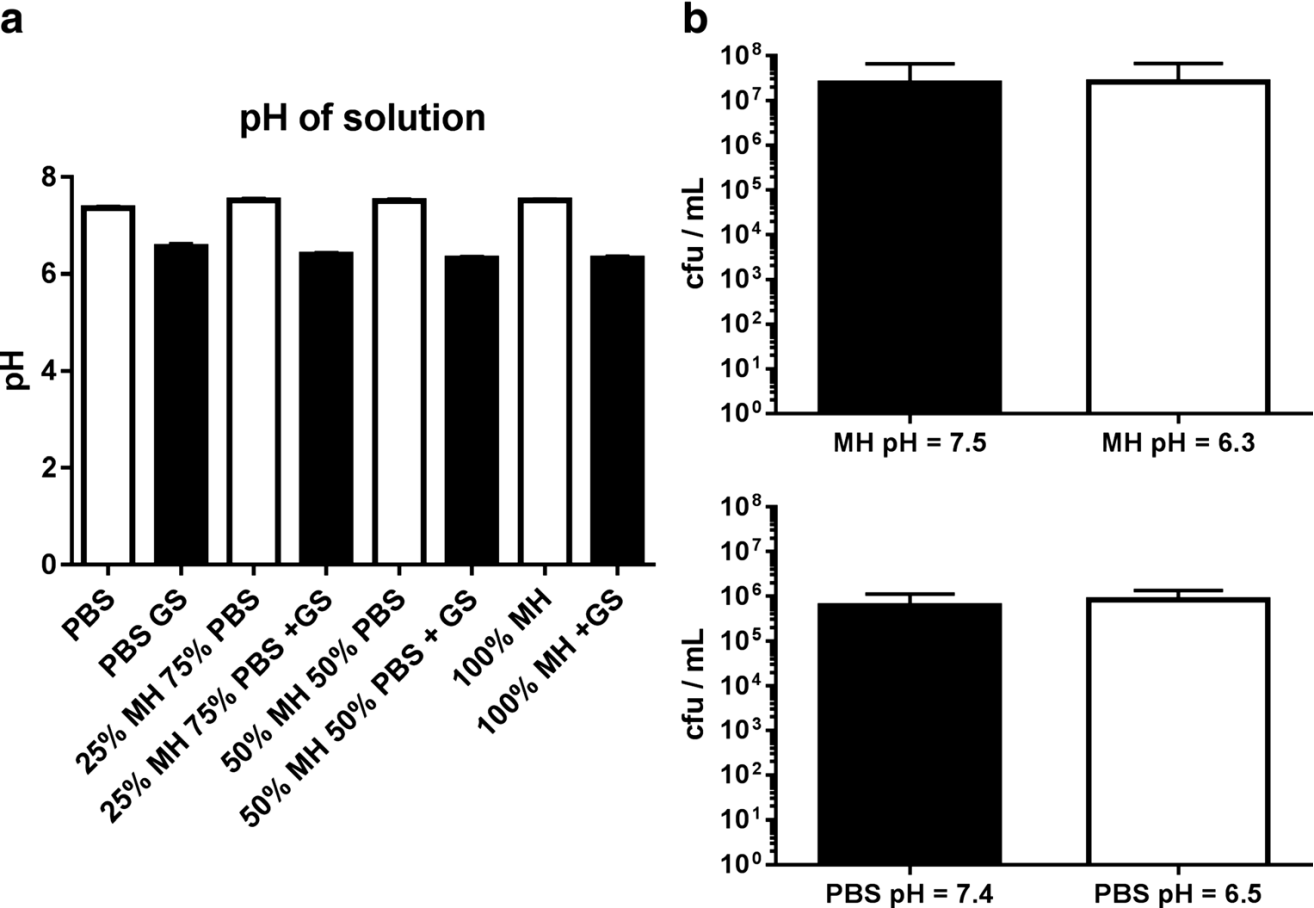
Differences in pH and the effect on survival of *Staphylococcus epidermidis*. **a** pH values of solutions (PBS) and Müller Hinton (MH) media. Highest tested concentration of gentamicin (GS; 0.5 mg per mL) is shown. **b** Survival of bacteria in a 48-h biofilm after incubation in PBS (top panel) or medium (lower panel) under pH values as induced by gentamicin sulfate for 24 h

Due to its acidic nature, gentamicin sulfate (GS) potentially reduces the pH value of solutions. We therefore determined the change in pH value caused by the highest concentration planned to be used in the upcoming experiments, 0.5 mg per mL gentamicin base, added to PBS, Müller Hinton medium, and dilutions thereof. GS significantly reduced the pH value of PBS from a mean of 7.36 to 6.56; the pH value of the used Müller Hinton medium was reduced from 7.51 to 6.32 in average. The pH values of the dilutions of MH and PBS and the stock solutions varied by numerically small amounts within one group (no gentamicin 7.36 to 7.52 and + 0.5 mg gentamicin per mL 6.32 to 6.56) (Fig. 2a).

Next, we performed survival tests to determine how this reduction in pH would influence bacterial viability. We adjusted the pH value of PBS and Müller Hinton by addition of sulfuric acid to the values determined for the addition of GS. The acidification of media did not significantly influence the survival of the tested 48-h *Staphylococcus epidermidis* biofilms; 609,200 ± 226,999 cfu/mL were detected at pH = 7.4 and 824,000 ± 230,773 cfu/mL at pH = 6.5 in PBS (*p* = 0.5256, Fig. 2b). Using Müller Hinton medium, 2,440,000 ± 1,901,000 cfu/mL were detected at pH = 7.5 and 2,620,000 ± 1,853,000 cfu/mL were detected at pH = 6.3 (*p* = 0.9476, Fig. 2b). Again, we did not observe a significant difference in survival comparing the values derived from incubation in the original stock solutions (MH vs. PBS, *p* = 0.2461).

### Survival of *Staphylococcus epidermidis* biofilms in the presence of gentamicin and under nutrient deprivation

We tested the survival of *Staphylococcus epidermidis* in established biofilms when incubated with rising concentrations of gentamicin under different nutrient saturations. We compared a laboratory strain (ATCC 12228) to four strains derived from patients with prosthetic joint infection. These four patient isolates have been determined to be resistant to gentamicin according to EUCAST disc diffusion testing.

**Fig. 3:**
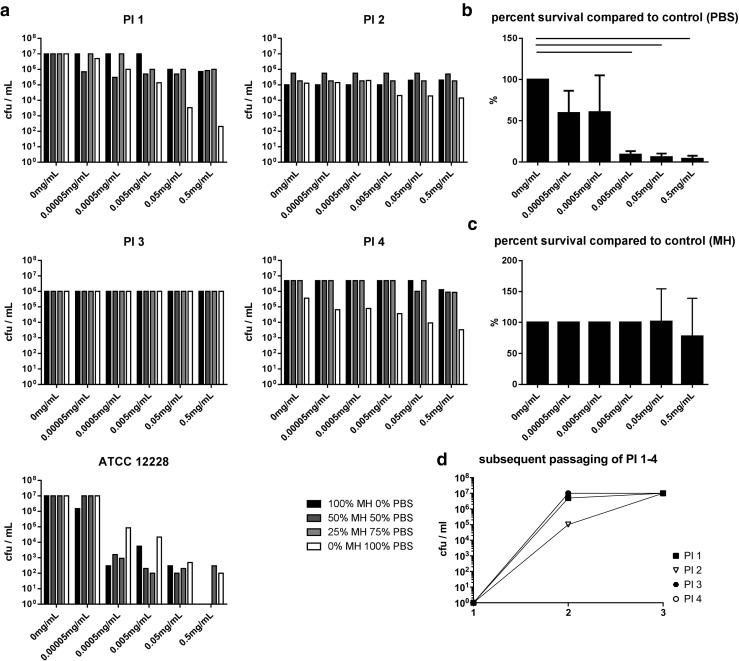
Survival of 48-h *Staphylococcus epidermidis* biofilms. **a** Survival in varying growth medium concentrations in presence of rising gentamicin concentrations. **b** Pooled data of responding PI strains (1, 2, 4) showing dose-dependent gentamicin response of survival in PBS. **c** Pooled data of responding PI strains (1, 2, 4) incubated in MH + 0.5 mg/mL gentamicin. **d** Growth curve of all PI in consecutive passaging (passages 1, 2, and 3)

The ATCC strain showed a dose-dependent reduction in survival across all tested media concentrations (approx. 7 log10 cfu/mL in pure MH to 0–2 log10 cfu/mL under 0.5 mg gentamicin per mL). One patient isolate (PI 3) did not show reduced survival by the addition of GS under any nutrient saturation (approximately 6 log10 cfu/mL under all conditions). The three other patient isolates tested here showed a dose-dependent reduction of bacterial survival when incubated in lowered nutrient saturations. This reduction was numerically high (up to 5 log10) and not detected when incubated with full medium (Fig. 3a). We calculated to what percentage the bacteria in the biofilm survived GS treatment in PBS compared to the no antibiotic control and found a statistically significant reduction of survival under three of the five tested concentrations (0.5 mg gentamicin per mL to 0.005 mg gentamicin per mL; *p* = 0.0391) (Fig. 3b). We could not detect a parallel reduction by GS treatment in the cultures incubated with full medium (*p* = 0.9944, Fig. 3c).

We examined an overall reduced growth in patient isolate 2 under all the tested growth conditions (5 log10 compared to approx. 7 log10 in the other three PI). To determine if this accounts for a general growth inhibition or an adaptation to the in vivo situation, we consecutively passaged all strains in vitro and determined if daughter colonies can develop the same growth potential under optimal growth conditions across all tested strains. We found that all isolates grew to equal numbers after consecutive passaging (approx. 7 log10, Fig. 3d).

## Discussion

We here describe that biofilms of *Staphylococcus epidermidis* strains which have been tested to be GS resistant under optimal growth conditions show a significantly reduced tolerance towards GS in low nutrient saturations.

We investigated the growth potential of *Staphylococcus epidermidis* ATCC 12228 and four patient-derived *Staphylococcus epidermidis* isolates derived from infected joint prosthesis in varying growth conditions. Mimicking a clinical situation, the nutrient saturation was reduced by diluting full medium with PBS.

We first tested the survival of biofilms of *Staphylococcus epidermidis* in low nutrient situations and found that the biofilms sustained even the complete lack of external nutrients during incubation with PBS. Unrelated experiments in our laboratory already showed that *Staphylococcus epidermidis* is highly capable of survival in low nutrient saturation since live bacteria could be derived from cultures grown for 12 months without addition of nutrients to the original solution (data not shown).

The acidic nature of gentamicin sulfate suggested a reduced pH value of the prepared solutions. We therefore measured the pH values that were achieved by addition of GS to PBS, Müller Hinton medium, and dilutions thereof. We tested the highest GS concentration used in our survival experiments since this presumably resulted in the highest change in pH of the solution. We detected a significant reduction of the pH value by addition of GS in all solutions. The reduction was slightly different comparing PBS to MH (7.4 to 6.5 and 7.5 to 6.3, respectively) showing a higher buffering capacity of PBS.

To determine if the reduced pH of the nutrient solutions would result in altered survival of the bacterial biofilm, we adjusted the pH of stock solutions with sulfuric acid. We used sulfuric acid instead of hypochloric acid to avoid the passaging of antibacterial chlorine into our test system. The reduced pH of the nutrient solution did not influence the survival of bacteria, neither under full medium nor in PBS. We observed a slightly (approx. 3-fold) higher rate of survival in MH compared to PBS, and this was however not statistically significant. Fux et al. (2005) in detail reviewed the genetic background of *Staphylococcus* leading to the expression of stress response genes, which in turn enable the bacteria to overcome periods of malnutrition and suboptimal pH. Less pronounced biofilm formation in medium with a low pH value has been shown by Chaieb et al. (2007), and the tested pH values however were far more acidic (pH = 5 and pH = 3) than the ones used in our experiments.

According to our results generated thus far, the nutrient saturation and the pH value of the solutions we planned to use in our upcoming experiments had no influence on basic bacterial survival in our model system.

Generally, we were interested in the effects a low nutrient situation, as can be found in clinical situations, e.g., after prosthetic joint surgery, would have on antibiotic susceptibility of bacteria in a biofilm. Testing of resistance against various antibiotics in the bacteriology laboratory is typically performed in optimal growth conditions provided by Müller Hinton medium/agar and incubation at 37 °C. Gentamicin functions via blocking the 30s ribosomal unit in bacteria, which is not existent in mammals. This block of the bacterial ribosome stops protein translation and ultimately abolishes bacterial survival. Henry-Stanley et al. (2014) recently showed that incubation of *Staphylococcus aureus* biofilms with gentamicin was ineffective at 5 μg/mL in 3× tryptic soy broth (TSB), while it did eradicate the biofilm at 1× TSB concentration and a one third dilution of TSB. This effect could not be observed with membrane-active antibiotics ampicillin and vancomycin (Henry-Stanley et al. 2014), suggesting an underlying metabolic mechanism. Addition of sugars and salts to tryptic soy broth and the one third dilution could not mimic the effects of an overall higher nutrient concentration (3× TSB).

Generally, biofilms deprived of nutrients become more resistant to antibiotic substances, which is explained by the low penetration of antibiotics through the extracellular matrix and the reduced metabolism of bacteria in a biofilm (Brown et al. 1988; Høiby et al. 2010).

During our study, we incubated 48-h biofilms of one ATCC and four patient isolates of *Staphylococcus epidermidis* biofilms in full Müller Hinton medium, 75% MH medium, 50% MH medium, and pure PBS. A lower nutrient concentration is suggested for bacterial niches in vivo, and our experimental setup was designed to replicate the situation which the patient isolates were originally derived from, an infected prosthetic joint (Tagil and Aspenberg 1998; Duffy et al. 2007). We added 10-fold serial dilutions of GS to the system resulting in final concentrations of 0.00005 to 0.5 mg/mL gentamicin and incubated biofilms for 24 h at 37 °C under a saturated water vapor atmosphere to avoid uneven evaporation of the medium, which could result in altered final concentrations of the administered antibiotic. The ATCC strain was determined to be susceptible to GS since the number of survivors dropped in a GS dose-dependent manner in all tested nutrient saturations. Generally, the lower concentrations of nutrients rendered the ATCC strain less susceptible to GS; live bacteria could still be recovered in the 0.5 mg gentamicin per mL group in the 25% MH and 100% PBS group while no surviving bacteria were found in the 100 and 50% Müller Hinton groups. This reflects the standard finding of a reduced metabolism caused by starvation leading to general tolerance towards antibiotics.

Reconfirming the gentamicin resistance of the patient isolates, only a slight and statistically not significant reduction in viable bacteria in the highest used doses of GS for two of four patient isolates was observed (0.5 mg gentamicin per mL for PI 1, 0.5 and 0.05 mg gentamicin per mL for PI 4). No effect could be achieved by GS on the other two strains in full medium.

One of the four tested patient isolates (PI 3) did not react to the addition of GS under any of the tested nutrient concentrations. Three strains however showed a significant and dose-dependent reduction of survival in the presence of GS when incubated in PBS (Fig. 3a). In contrast to the Henry-Stanley group, who added salts and sugars to the solutions containing less nutrients (Henry-Stanley et al. 2014), we added protein (1% BSA or FBS, respectively) to the PBS-gentamicin solution and detected a further, but statistically not significant reduction in surviving bacteria in subsequent experiments (approx. 0.5 log10, data not shown). We think that addition of protein temporarily boosted the bacterial metabolism leading to inactivation of the affected bacteria. The use of gentamicin, which blocks the bacterial protein translation system, might highlight the effects of adding fresh protein as source of amino acids.

Patient isolate 2 generally grew to numbers between 1 and 2 log10 lower compared to the other patient isolates. Interestingly, this isolate also showed the lowest reduction of growth by GS in PBS. It thus seemed that the observed effect was related to in vivo adaptations regarding metabolic activity or stress response. The low colony counts established by this strain in the original biofilm did not reflect a general block in reproduction capability but rather an adaptation to the in vivo situation, since subsequent in vitro passaging in optimal growth conditions (passages 1–3) of a single colony led to equal numbers of colony-forming units per milliliter in the daughter colonies across all tested strains (Fig. 3d).

In conclusion, we here show that susceptibility to gentamicin sulfate relates to the provided nutrient saturation. Standard testing is performed under optimal growth conditions. Four bacterial strains isolated directly from the patient built biofilms and were resistant to GS when incubated in full medium. Supplied with only limited to no external nutrients, three of the four strains showed to be sensitive to GS in a dose-dependent manner.

We believe the data presented here provide significant evidence that bacterial strains adapted to in vivo situations can display a different phenotype including tolerance to antibiotics when derived directly from the patient compared to the same strain grown and maintained under laboratory settings. This can relate to a highly adapted stress response evolved in the biofilm over long incubation times in the patient. Thus, testing and profiling of patient-derived bacteria should be performed under conditions similar to those found in the niche occupied by the bacteria in vivo.
